# A Rare Novel *CLCN2* Variation and Risk of Gilles de la Tourette Syndrome: Whole-Exome Sequencing in a Multiplex Family and a Follow-Up Study in a Chinese Population

**DOI:** 10.3389/fpsyt.2020.543911

**Published:** 2020-12-03

**Authors:** Aihua Yuan, Zengge Wang, Wen Xu, Qiang Ding, Ying Zhao, Jingjing Han, Jinhua Sun

**Affiliations:** ^1^Shanghai Mental Health Center, School of Medicine, Shanghai Jiao Tong University, Shanghai, China; ^2^Beijing Key Laboratory for Genetics of Birth Defects, Beijing Pediatric Research Institute, Beijing, China; ^3^Genetics and Birth Defects Control Center, National Center for Children's Health, Beijing, China; ^4^Beijing Children's Hospital, Capital Medical University, Beijing, China; ^5^Department of Psychological Medicine, Children's Hospital of Fudan University, Shanghai, China

**Keywords:** Gilles de la Tourette syndrome, whole-exome sequencing, CLCN2, rare variation, multiplex family

## Abstract

Rare inherited variations in multiplex families with Gilles de la Tourette syndrome (GTS) are suggested to play an important role in the genetic etiology of GTS. In order to explore the rare inherited variations with the risk of GTS, whole-exome sequencing (WES) was performed in a family with three affected patients with GTS. Among the five novel rare variations identified by WES, *CLCN2* G161S was presented in three patients, but not in four unaffected individuals, and thus co-segregated with GTS. A validation study was also performed in a cohort of Chinses Han population to further examine the identified rare variants. *CLCN2* G161S was genotyped in 207 sporadic patients with tic disorder including 111 patients with GTS and 489 healthy controls. Compared with that in controls [allele frequency (AF) = 0], *CLCN2* G161S had higher variant AF in patients with tic (AF = 0.00483) and in patients with GTS (0.00900), respectively. However, this variant was absent from the current 1000 Genome databases, and the variant AF is very low in the current public databases including ExAC (AF = 0.00001) and gnomAD (AF = 0.00003). Our results suggest that *CLCN2* G161S might play a major role in the genetic etiology of GTS, at least in a Chinese Han population.

## Introduction

Gilles de la Tourette syndrome (GTS), also known as Tourette's syndrome (TS) or Tourette's disorder, is a common, heritable neurological disorder manifested by chronic motor and vocal tics that persist for more than 1 year with childhood onset. The global prevalence of GTS ranges between 0.3 and 1% (1).

GTS is a complex disorder, and genetics plays a critical role in the pathogenesis of this disorder. From family studies, it has been determined that first-degree relatives of affected individuals are at 5–15-fold increased risk of GTS compared to the general population (2). In twin studies, the heritability has been estimated at 70–80%, which is one of the highest heritability for a neuropsychiatric disorder (3). Association studies and linkage analyses of GTS conducted to date have led to identification of some risk genes relevant to neuronal outgrowth and neurotransmitter systems, such as genes encoding SLITRK1 (Slit and Trk-like 1), a member of a neuronal transmembrane protein family, serotonin receptors, and dopamine receptors, none of which have been consistently replicated (4). With the advent of the next-generation sequencing technology, rare variant studies using whole-exome sequencing (WES) might be useful for identifying susceptibility genes in complex neuropsychiatric diseases (5). A recent GTS study of *de novo* variation using WES found 25 *de novo* coding variants in 45 samples from 15 trios (6). A study examining rare inherited variants in multiplex families with GTS identified a rare nonsense mutation in *PNKD* that co-segregated with the phenotype of the disorder, which resulted in reduced expression of *PNKD* in neurons derived from individuals with GTS. Another GTS study of rare inherited variants identified three novel rare variations in *MRPL3, DNAJC13*, and *OFCC1* that segregated with chronic tic disorder phenotype in a three-generation pedigree with seven family members showing GTS symptoms (7).

In order to further investigate the potential rare variants with large effect sizes in patients with GTS, WES was performed on the individuals of a multiplex family with GTS. Furthermore, it is also critical to confirm whether the candidate risk variants in a multiplex family identified by WES are involved in the genetic etiology of the disease using independent samples. Therefore, a follow-up validation study was performed in a Chinese Han population.

## Materials and Methods

### Participants

**Figure 1:**
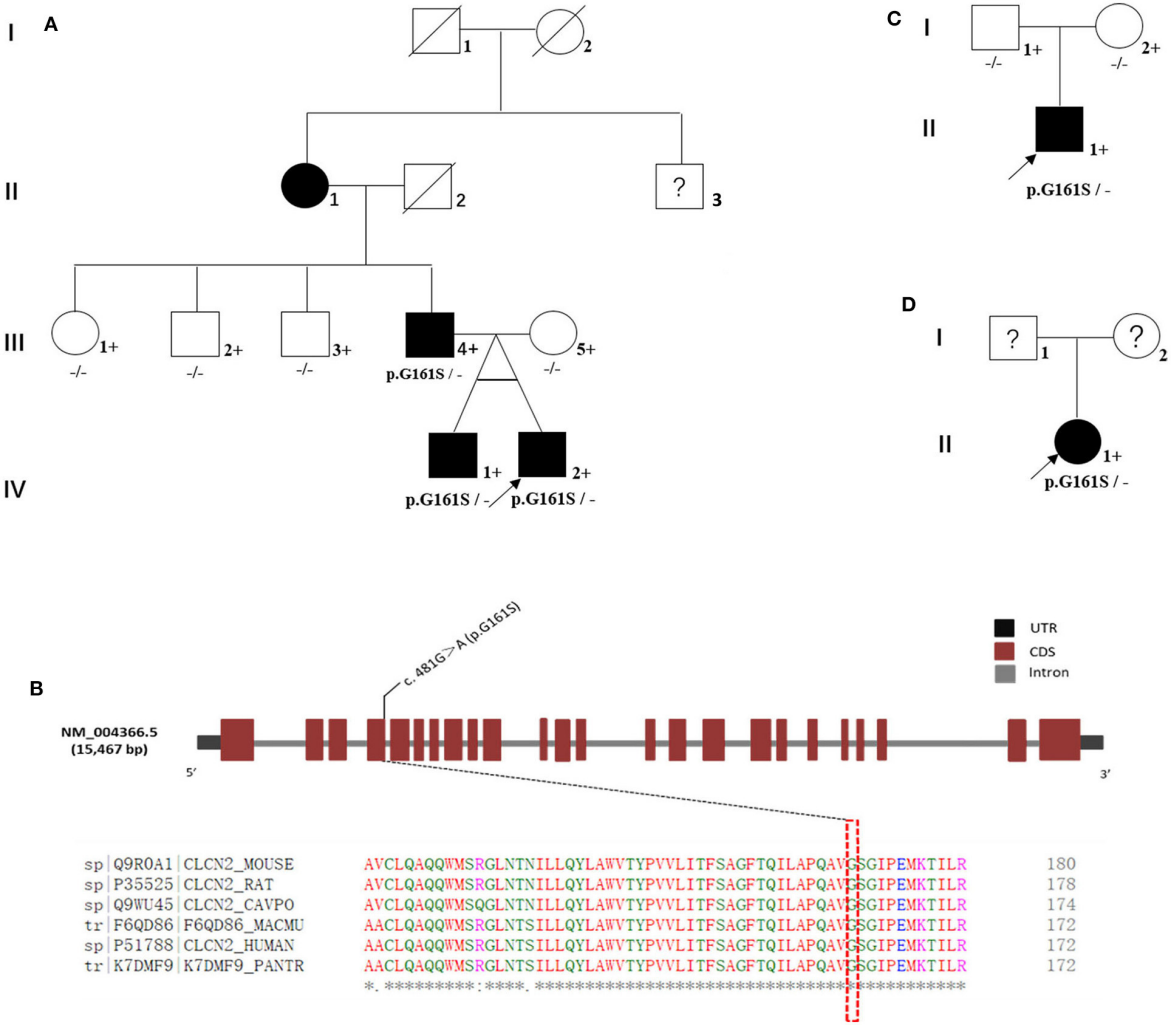
Familial cohorts with Tourette syndrome. **(A)** Family 1^#^: a multiplex family with Tourette syndrome; three affected individuals were G161S heterozygous (III-4, IV-1, and IV-2). **(B)** *CLCN2* G161S is located in highly conserved regions in multiple species. **(C)** Family 2^#^: the biological parents of the proband carried the wild-type allele. *CLCN2* G161S was a *de novo* mutation in this family. **(D)** In family 3^#^, the biological parents of the proband could not be reviewed, and the DNA samples were unavailable. Whether *CLCN2* G161S was a *de novo* mutation in this family remained undetermined. Black-shaded symbols represent GTS-affected individual, unfilled symbols represent unaffected individuals, and question marks represent individuals with unknown disease status. Crosses represent individuals with available genomic DNA samples. Proband is marked with an arrow.

In the multiplex family (named family 1^#^, Figure 1A), the proband was a boy. He, his monozygotic twin brother, and his father were diagnosed with GTS (Figure 1A: IV-1, IV-2, and III-4). The proband's grandmother had been diagnosed with tic disorder (Figure 1A: II-1). The diagnosis of tic or GTS (as a subtype of tic disorder) was made by an experienced deputy chief psychiatrist according to the *Diagnostic and Statistical Manual of Mental Disorders*, fourth edition, revision version (DSM-IV-TR) criteria made by the American Psychiatric Association (APA). The MINI-International Neuropsychiatric Interview (MINI), a semi-structured interview questionnaire, was conducted to meet the demand for a short but accurate structured psychiatric interview. The proband's mother, aunt, and uncles were confirmed as unaffected by an experienced psychiatrist using an unstructured interview (Figure 1A: III-5, III-1, III-2, and III-3). We were unable to obtain blood samples from several family members including the proband's grandmother and his grandmother's brother. Their current mental status was designated as unknown and needs to be confirmed through the formal interview, although the proband's father said his mother (the proband's grandmother) had only a few symptoms such as blinks and shrugs and the grandmother recalled that she often had loud throat vocalization. Rediagnosis of the proband's grandmother and diagnosis of the grandmother's brother were hindered by living in a remote rural location in China.

In the follow-up validation study, the cohort consisted of 207 patients with tic disorder (170 males and 37 females, mean age 10.6 [SD 3.6] years), including 111 patients with GTS (91 males and 20 females, mean age 11.4 [SD 3.2] years), and 489 healthy control individuals (296 males and 193 females, mean age 16.0 [SD 9.7] years) (Table 1). The patients and control groups were not sex or age matched. All patients with tics were subjected to psychiatric assessment. Specifically, patients were diagnosed according to DSM-IV-TR criteria by APA for tic disorder or GTS. Healthy controls were provided by the Women and Children's Hospital of Xiamen University and were assessed with a self-edited questionnaire in order to exclude psychiatric disorders and major organic diseases. Subjects were excluded if there were three generations of family members with mental illness.

**Table 1:** Demographics of participants and clinical evaluation of patients.

**Characteristics**	**TD (*N* = 207)**	**GTS (*N* = 111)**	**HC (*N* = 489)**
Age (years: mean ± SD)	10.6 (3.6)	11.4 (3.2)	16.0 (9.7)
Male gender (*n*, %)	170 (82.1)	91 (81.9)	296 (60.5)
**YGTSS score**			
Motor tic severity	14.2 (4.7)^a^	11.1 (7.5)^b^	/
Vocal tic severity	8.4 (7.0)^a^	10.2 (6.7)^b^	/
Total tic severity score	22.6 (9.5)^a^	25.1 (9.1)^b^	/
Functional impairment	24.1 (12.7)^a^	27.0 (13.5)^b^	/
Total YGTSS score	46.4 (19.3)^a^	51.8 (19.1)^b^	/

*TD, tic disorder; GTS, Gilles de la Tourette Syndrome; HC, healthy controls; YGTSS, Yale Global Tic Severity Scale*.

a *Data available for 147 TD subjects*.

b *Data available for 83 GTS subjects*.

This study was approved by the Ethics Committee of the Children's Hospital of Fudan University. Written informed consent was obtained from all participants and/or their families. All participants were of Han Chinese descent.

### WES Study

The entire sequencing procedure was conducted. In brief, 3 μg of DNA extracted from peripheral blood samples was sheared through a Covaris M220 Ultrasonicator (Covaris, Woburn, MA, USA) to create 150–200-bp fragments. The adapter-ligated library was prepared with a SureSelectXT Library Prep Kit, and the capture library was constructed using a SureSelectXT Human All Exon V6 Kit (Agilent Technologies, Santa Clara, CA, USA) to enrich the coding exons and flanking intronic regions. Clusters were generated via isothermal bridge amplification and sequenced on the HiSeq 2000 system (Illumina, San Diego, CA, USA). Quality assessment of base calling and sequence reads was performed using the Illumina Sequence Control Software v4.0.4 with Real-Time Analysis. NextGENe® v2.4.1 software (SoftGenetics, LLC, State College, PA, USA) was used to align the sequence reads with the reference human genome (Human GRCh 37.3, http://hgdownload.soe.ucsc.edu/goldenPath/hg19/snp135Mask/). All acquired single-nucleotide variants (SNVs) and indels were saved in VCF file format and uploaded on Knowledge-Driven NGS Analysis-TGex™ (LifeMap Sciences, Inc., Alameda, CA, USA) for detailed filtering and interpretation.

To prioritize variations, we applied numerous filtering steps to variations in WES (Table 2). First, we filtered out variations with <20X coverage. Further, we filtered out variations whose population frequency was <0.1% in the gnomAD_EAS. Finally, the likely pathogenic (LP) and pathogenic (P) variations were selected according to the scoring criteria of the American College of Medical Genetics and Genomics (ACMG).

**Table 2:** Filtering steps to variations identified by WES for the multiplex GTS family.

**Filtering criteria**	**Number of remaining variations**
Total variations identified	30,534
Covered with ≥20 reads	23,658
Frequency in gnomAD_EAS ≤ 0.1%	1,764
LP/P according to the ACMG	5

*WES, whole-exome sequencing; GTS, Gilles de la Tourette Syndrome; LP, likely pathogenic; P, pathogenic; ACMG, American College of Medical Genetics and Genomics*.

To assess co-segregation of prioritized variations, Sanger sequencing was performed for the individuals of the multiplex family including the proband's aunt and two uncles. PCR was carried out on a T100 Thermal Cycler (Bio-Rad, Inc. Hercules, CA, USA), and the resulting products were sequenced using the ABI 3130xl sequencer (Thermo Fisher Scientific, Inc., Waltham, MA, USA) with primers. The sequencing data were analyzed using Mutation Surveyor DNA Variant Analysis software v4.0 (SoftGenetics, LLC, State College, PA, USA). Primer sequences and detailed information of amplification conditions are available upon request.

### Validation Study

To determine whether the rare splicing *CLCN2* variation prioritized in WES of the multiplex family contributed to the genetic etiology of GTS, *CLCN2* G161S was verified using an independent population comprising 207 sporadic patients with tic disorder and 489 healthy controls from the Women and Children's Hospital of Xiamen University. *CLCN2* G161S was genotyped using MALDI-TOF mass spectrometry (MS), as previously described (8). The *CLCN2* primers used were as follows: 5′-ACGTTGGATGATCACTTTCTCAGCCGGATTC-3′ (forward) and 5′-ACGTTGGATGATTCGATGCACCCATTTCAGG-3′ (reverse). To remove the remaining dNTPs in the PCRs, we performed shrimp alkaline phosphatase treatment followed by primer extension. The sequence for the extension primer was *CLCN2*_U: CCTGCCCCTCAGGCTGTC. The extension products were desalted for MS analysis, as previously described. Data acquisition from the SpectroCHIP was performed using Bruker Compact MALDI-TOF MS (Bruker Daltonics), and data analyses were carried out with the TyperAnalyzer application, version 4.0 (Sequenom). All primers were purchased from Integrated DNA Technologies. All other reagents were purchased from Sequenom.

Deviations from the Hardy–Weinberg equilibrium were tested using the χ^2^ test for goodness of fit. Allelic association of variants was tested using Fisher's exact test. Allele frequencies (AFs) were estimated in all samples using the SHEsisPlus program [http://shesisplus.bio-x.cn/SHEsis.html (9)].

## Results

WES was performed for a multiplex family (family 1^#^) including the proband, his monozygotic twin brother, and the proband's father and mother and captured almost all regions (59.04 Mb) in each exome (Supplementary Table 1). The average read depth varied from 108.48X to 123.00X, and 94.10 to 96.00% of target regions were covered by 20 or more reads. A total of 30,534 sequence variations were called from WES in the multiplex family (Table 2). After several filtering steps, we prioritized four rare missense variations (*C2CD2L* R497W, *MSH4* E379K, *TULP4* S1451I, and *UTP4* R634W) and a rare splicing variation (*CLCN2* G161S). These variations were confirmed by Sanger sequencing. These five rare variants were predicted to be “probably damaging” by PolyPhen-2 (http://genetics.bwh.harvard.edu/pph2/) and Sorting Intolerant from Tolerant (SIFT: http://sift.bii.a-star.edu.sg/index.html). Among these five variants, *CLCN2* G161S was identified in three affected individuals (Figure 1A: III-4, IV-1, and IV-2), but not in one unaffected individual (Figure 1A: III-5; Figure 1B), which was verified by Sanger sequencing (Supplementary Figure 1). In contrast, the other four variations were present in both affected and unaffected individuals (Table 3). In order to investigate whether *CLCN2* G161S co-segregates with the phenotype of GTS, *CLCN2* G161S was examined in three unaffected relatives including the proband's aunt and two uncles by Sanger sequencing. The results showed that the proband's aunt and uncles carried the wild-type allele. Therefore, *CLCN2* was considered to be the most promising candidate gene for GTS.

**Table 3:** Rare variation prioritized by WES for the GTS multiplex family.

**Chromosome**	**Position^a^**	**Gene**	**Protein**	**Allele^b^**	**PolyPhen-2/SIFT**	**Family member (available DNA sample)**		**Transmission**
						**Affected (*n* = 3)**	**Unaffected (*n* = 4)**	
3	1.84E+08	*CLCN2*	G161S	C/T	Damaging	3	0	Father
11	1.19E+08	*C2CD2L*	R497W	C/T	Damaging	2	2	Mother
1	76,288,239	*MSH4*	E379K	G/A	Damaging	1	1	/
6	1.59E+08	*TULP4*	S1451I	G/T	Damaging	1	1	/
16	69,201,044	*UTP4*	R634W	C/T	Damaging	2	1	/

*WES, whole-exome sequencing; GTS, Gilles de la Tourette syndrome; SIFT, Sorting Intolerant from Tolerant*.

a *Position according to GRCh 37*.

b *Referenced/mutation allele*.

**Figure 2:**
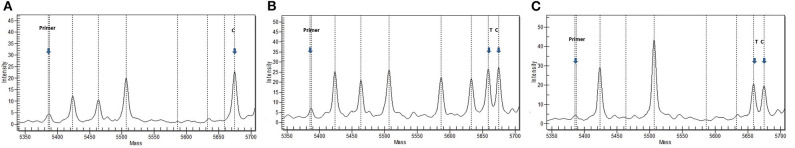
A representative mass spectrum of the screening sample. **(A)** Wild-type mass spectrum for a patient with GTS. **(B)** Mutation mass spectrum for a patient with GTS. **(C)** Mutation mass spectrum for a patient with GTS. Primer, unextended primer; T, extension products for the mutant sequence; C, extension products for the wild-type sequence C.

Subsequently, a validation study was performed to assess the association of *CLCN2* G161S with tic disorder in an independent population. We genotyped *CLCN2* G161S in 489 healthy controls and 207 sporadic patients with tic disorders including 111 GTS patients. In sporadic patients, a heterozygous *CLCN2* G161S was identified in a male patient with GTS and attention deficit hyperactivity disorder (ADHD) and a female patient with GTS and obsessive–compulsive disorder (OCD), and the 489 controls all carried the wild-type allele (Figure 2). Compared with that in controls (AF = 0), *CLCN2* G161S had a higher variant AF in patients with tic (AF = 0.00483) and in patients with GTS (AF = 0.00900). No deviation from the Hardy–Weinberg equilibrium was found in genotype distribution of the variant. No significant association was detected between *CLCN2* G161S and tic disorder (*P* = 0.029, adjusted *P* = 0.999) and *CLCN2* G161S and GTS (*P* = 0.003, adjusted *P* = 0.996) after age and sex correction (Table 4).

**Table 4:** Genotyping of *CLCN2* G161S in the follow-up study.

**Sample**	**Variation**	**Patient**				**Control**				***P***	***P^a^***	**OR**	**95% CI**
		**C/T**	**C/C**	**T/T**	**MAF**	**C/T**	**C/C**	**T/T**	**MAF**				
Patients with tic (*n* = 207)	*CLCN2* G161S	2	205	0	0	0	489	0	0	0.03	1	-	-
Patients with GTS (*n* = 111)	*CLCN2* G161S	2	109	0	0.01	0	489	0	0	0	1	-	-

*WES, whole-exome sequencing; MAF: mutant allele frequency; OR, odds ratio*.

a *P-values are adjusted for age and sex*.

In addition, in order to identify whether the *CLCN2* G161S carried by the two sporadic GTS patients was inherited from their parents, the proband's parents (family 2^#^) were genotyped using Sanger sequencing and were found to carry the wild-type allele (Figure 1C). Therefore, *CLCN2* G161S was a *de novo* mutation in family 2^#^. In family 3^#^, the biological parents of the proband could not be reviewed, their disease status was unknown, and DNA samples were unavailable (Figure 1D). Therefore, whether *CLCN2* G161S was a *de novo* mutation in family 3^#^ remained undetermined.

The following are the clinical descriptions of the proband and his monozygotic twin brother with GTS in family 1^#^. The proband was a 12-year-old boy and was a younger monozygotic twin brother. He had many severe symptoms for more than 3 years including blinking, shaking head, shrugging, plucking up the abdomen, and shrinking the nose repeatedly, accompanied by repeated throat voice for more than 1 year. The total score of the Yale Global Tic Severity Scale (YGTSS) was 64, which indicated that the severity of his tics was high. Brain structure examination was performed using a 3.0-T magnetic resonance imaging (MRI) instrument, which showed a normal brain structure. The results of electroencephalogram (EEG) examination and biochemical and immunological examinations of peripheral blood were also normal (Table 5).

**Table 5:** Clinical phenotypes of the five individuals heterozygous for *CLCN2* G161S.

**Clinical phenotype**	**Family 1**^****#****^			**Family 2^**#**^**	**Family 3^**#**^**
	**Father**	**Younger monozygotic twin brother**	**Elder monozygotic twin brother**	**Proband**	**Proband**
Transmission		Paternal	Paternal		
Sex	Male	Male	Male	Male	Female
Age	45Y	12Y3M	12Y3M	11Y4M	12Y
DSM-IV-TR diagnosis	GTS	GTS	GTS	GTS	GTS
Comorbidity (mental disorder)	No	No	No	ADHD	OCD
Illness duration	26Y	5Y	7Y	4Y	3Y
**YGTSS score**					
Motor tic severity	5	15	5	15	15
Vocal tic severity	6	19	15	11	5
Functional impairment	10	30	20	30	20
Total YGTSS scores	21	64	40	56	40
Past history	Nasal sinusitis	Hernia	No	No	Language and motor retardation
Brain MRI/EEG	Null	Normal	Normal	Normal	Normal
**Laboratory tests**					
Rheumatoid factor (RF, IU/ml)	Null	Null	Null	<8.88	<10.1
Antistreptolysin O (anti-O, IU/ml)	Null	<13.1	Null	<13.2	244↑
Erythrocyte sedimentation rate (ESR, mm/h)	Null	7	Null	27↑	7
Blood ceruloplasmin (g/L)	Null	0.223	Null	0.25	0.19↓

*GTS, Gilles de la Tourette syndrome; ADHD, attention deficit/hyperactivity disorder; OCD, obsessive–compulsive disorders; YGTSS, Yale Global Tic Severity Scale; MRI, magnetic resonance imaging; EEG, electroencephalogram; Null, no information or unfinished; Y, years; M, months*.

The elder monozygotic twin brother developed tic symptoms earlier than the proband, and his age of onset was 5 years old according to his mother's recollection. The first episode of symptoms included repeated blinking of the eyes. After 6 months of treatment, the symptoms improved. However, 2 years later, the blink symptoms recurred, and other tic symptoms also occurred intermittently, such as throat vocalization, sniffing, and sound from the nose. Although his age at onset was earlier than that of the proband, he still had a variety of tic symptoms, which were relatively less severe than that of the proband. The total score of YGTSS was 40, which indicated that his tic severity was moderate. His mother was more concerned about his younger brother's tics.

The proband of family 2^#^ was an 11-year-old boy. He had a variety of twitching symptoms since the first grade of primary school, such as shrugging his shoulders, grinning, making voices in his throat, and liking to spit. In addition to twitching symptoms, he exhibited inattention symptoms in class and procrastination toward his homework. He was also diagnosed with ADHD. His total YGTSS score was 56, indicating a severe level (Table 5).

The proband of family 3^#^ was a 12-year-old girl. She had a history of tics for more than 3 years. She twisted her neck, shook her limbs, blinked repeatedly, and smacked her lips back and forth to make a loud voice. Now she often blinks and shrugs repeatedly. The total YGTSS score was 40, indicating a moderate level. In addition, she had some OCD symptoms, such as writing repeatedly and modifying and counting numbers repeatedly although that was not what she wanted to do (Table 5).

## Discussion

In the present study, five rare variations were identified by WES in a multiplex family with GTS. Among these variations, only *CLCN2* G161S co-segregated with the phenotypes of GTS and was inherited from the proband's father. In the follow-up validation study, *CLCN2* G161S was identified in two sporadic patients with GTS. In family 2^#^, the unaffected parents of the proband carried the wild-type allele; therefore, *CLCN2* G161S was a *de novo* mutation in the family. In family 3^#^, whether *CLCN2* G161S was an inherited or a *de novo* mutation was unknown due to the unavailability of the sample of the proband's parents. In addition, our follow-up validation study showed that *CLCN2* G161S had a higher variant AF in sporadic patients with GTS (AF = 0.004) than in the controls (AF = 0). Interestingly, *CLCN2* G161S was absent from the current 1000 Genome databases, and the variant AF was very low in the current public databases including ExAC (AF = 0.00001) and gnomAD (AF = 0.00003). We screened the *CLCN2* gene for its relevance to neurodevelopmental disorders and found that *CLCN2* has been strongly implicated in neurological disorders.

Certain homozygous or heterozygous *CLCN2* mutations can cause two distinct phenotypes: leukoencephalopathy with ataxia (*CLCN2*; MIM#615651) and idiopathic generalized epilepsy (*CLCN2*; MIM#607628) including juvenile myoclonic epilepsy and juvenile absence epilepsy. For example, a homozygous 6-bp in-frame deletion (c.430_435del) was found in a woman from North Africa, and a homozygous 6-bp in-frame deletion (c.430_435del) and heterozygous R235Q and R577Q were identified in a sib of Tunisian origin with juvenile myoclonic epilepsy and in two German sibs with idiopathic generalized epilepsy (10, 11). Interestingly, a recent study showed that *CLCN2* G161S, as a *de novo* mutation, was found in three patients with childhood absence epilepsy (12). In this study, we identified the heterozygous *CLCN2* G161S variant in a multiplex family with GTS (family 1^#^). In this family, the variant carried by monozygotic twin brothers was inherited from their father. In family 2^#^, the *CLCN2* G161S variant, as a *de novo* mutation, was found in the patient but not his unaffected parents. Both patients with epilepsy and GTS had an early onset age. Therefore, we speculated that *CLCN2* G161S might cause a neurodevelopmental dysfunction underlying epilepsy and GTS. However, its functional implications remain to be clarified.

*CLCN2* encodes chloride channel 2 (CLC-2), which is a type of permeable chloride channel that belongs to the family of *CLCN2* channel/transport proteins (13). *CLCN2* is expressed in glia precursors during development and is required for their differentiation into astrocytes and oligodendrocytes (14, 15). Mutations in *CLCN2* are responsible for leukoencephalopathy with ataxia (16). Several lines of evidence suggest that oligodendrocyte dysfunction and white matter disconnection are involved in the pathophysiology of GTS (17, 18). Compared to healthy control, fractional anisotropy decreases and radial diffusivity increases in deep white matter tracts in cortico-striato-thalamo-cortical circuit as well as superficial white matter in GTS children; furthermore, lower fractional anisotropy values and higher radial diffusivity values in white matter regions are correlated with more severe tics (19). Taking these findings into account, we speculate that *CLCN2* G161S might elicit abnormal signal conduction by damaged myelin across different brain regions, which results in GTS development.

There were some limitations in our study. First, we prioritized the rare variations that were predicted to be “probably damaging” by PolyPhen-2 and SIFT. Thus, other rare variations might have been overlooked. Second, WES can detect exon variants but not promoter variants, which could contribute to GTS. Third, blood samples from the proband's grandmother were not available. As described by the proband's father, the proband's 82-year-old grandmother also showed symptoms of tic disorder; however, we did not obtain her peripheral blood sample for WES verification and needed face-to-face interviews to obtain more information supporting the GTS diagnosis or just tic disorder.

In conclusion, our study suggests that *CLCN2* G161S might play a major role in the genetic etiology of GTS, at least in the Chinese Han population.

## Data Availability Statement

According to national legislation/guidelines, specifically the Administrative Regulation of the People's Republic of China on Human Genetic Resources (https://www.gov.cn/zhengce/content/2019-06/10/content_5398829.html, https://english.www.gov.cn/policies/latest_releases/2019/06/10/content_281476708945462.html), no additional raw data is available at this time. Data of this project can be accessed after an approval application to the China National Genebank (CNGB, https://db.cngb.org/cnsa/). Please refer to https://db.cngb.org/, or email: CNGBdb@cngb.org for detailed application guidance. The accession code CNP0001372 should be included in the application.

## Ethics Statement

Written informed consent has been obtained from the minor(s)' legal guardian/next of kin for the publication of any potentially identifiable images or data included in this article.

## Author Contributions

JS, AY, and ZW contributed to the conception and design of the study. AY and ZW organized the database. AY and JS performed the statistical analysis. AY wrote the first draft of the manuscript. JS and ZW revised the manuscript. QD, WX, JH, and YZ were charged with the collection of clinical data and psychological assessment. All authors contributed to manuscript revision and read and approved the submitted version.

## Conflict of Interest

The authors declare that the research was conducted in the absence of any commercial or financial relationships that could be construed as a potential conflict of interest.

## Supplementary Material

Supplementary Figure 1 Confirmation of the missense mutation in family 1^#^ by Sanger sequencing. III-5 is G/G; III-4, IV-1 and IV-2 are G/A heterozygote. The red dotted line shows the difference.
