# Supplementary figures and images for: A Rare Novel CLCN2 Variation and Risk of Gilles de la Tourette Syndrome: Whole-Exome Sequencing in a Multiplex Family and a Follow-Up Study in a Chinese Population
Source: Front Psychiatry. 2020 Dec 3;11:543911. doi: 10.3389/fpsyt.2020.543911 (PMC7744286; doi:10.3389/fpsyt.2020.543911)

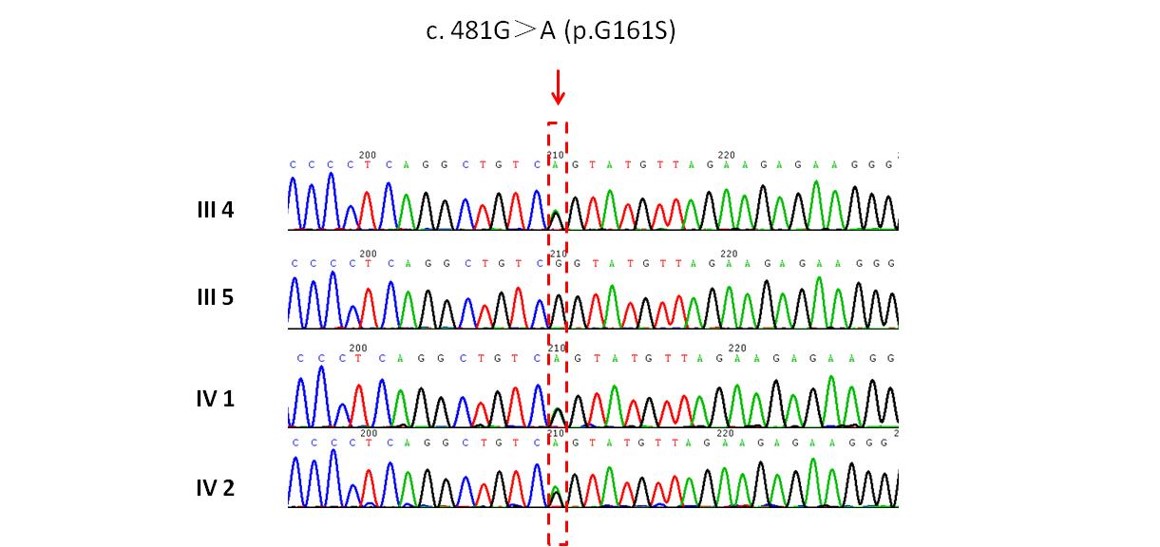

Supplement: Supplementary Figure 1 — Confirmation of the missense mutation in family 1# by Sanger sequencing. III-5 is G/G; III-4, IV-1 and IV-2 are G/A heterozygote. The red dotted line shows the difference. [file Image_1.JPEG]
